# A simple method for the determination of reduction potentials in heme proteins

**DOI:** 10.1016/j.febslet.2013.12.030

**Published:** 2014-03-03

**Authors:** Igor Efimov, Gary Parkin, Elizabeth S. Millett, Jennifer Glenday, Cheuk K. Chan, Holly Weedon, Harpreet Randhawa, Jaswir Basran, Emma L. Raven

**Affiliations:** a Department of Chemistry, Henry Wellcome Building, University of Leicester, University Road, Leicester LE1 7RH, United Kingdom; b Department of Biochemistry, Henry Wellcome Building, University of Leicester, University Road, Leicester LE1 9HN, United Kingdom

**Keywords:** Heme, Heme protein, Reduction potential, Redox

## Abstract

We describe a simple method for the determination of heme protein reduction potentials. We use the method to determine the reduction potentials for the PAS-A domains of the regulatory heme proteins human NPAS2 (*E*_m_ = −115 mV ± 2 mV, pH 7.0) and human CLOCK (*E*_m_ = −111 mV ± 2 mV, pH 7.0). We suggest that the method can be easily and routinely applied to the determination of reduction potentials across the family of heme proteins.

## Highlights

• A simple method for determination of heme protein reduction potentials is described.
• We use the method to determine reduction potentials for human NPAS2 and human CLOCK.
• The method can be easily applied to other heme proteins.

## 1 Introduction

Heme-containing proteins form a vast and biologically important superfamily: they are found in all living species and carry out a very wide array of functions. The most well studied examples are the O_2_ transport proteins (the globins), the electron transfer proteins (the cytochromes), and the various catalytic enzymes, the best characterized perhaps being the P450s, NO synthases and the heme peroxidases. But beyond the scope of these ‘traditional’ heme proteins, there is now clear evidence of a wider regulatory role for heme in biological systems; examples include roles for heme in ion channel regulation, transcriptional control and gas sensing, but these roles are as yet only poorly defined at a structural, mechanistic and functional level.

An important property of the heme group is the redox behavior of the metal, which can exist in +2, +3 or +4 oxidation states. Across the entire family of heme proteins, the stability of individual iron oxidation states can vary enormously as a complex function of different variables including the nature, orientation and hydrogen bonding interactions of the heme ligand(s), the identity of active site residues around the heme group, solvent accessibility, the heme substituents, the orientation of the heme group and vinyl groups, hydrogen bonding interactions of the heme propionates, and the extent of heme ruffling (see for example [1], [2], [3], [4], [5], [6], [7], [8], [9], [10], [11], [12], [13], [14]). The reduction potential of the heme group is a key determinant of biological function, and yet reduction potentials for heme proteins are not routinely reported, perhaps because the measurements have often depended on specialist or somewhat inaccessible methodologies (see for example [15], [16], [17], [18], [19], [20], [21], [22], [23], [24], [25], [26], [27]). In this paper, we describe a simple method for the determination of heme protein reduction potentials. The method allows the determination of the reduction potential from equilibrium concentrations and without the need for measuring a potential; it thus avoids some of the difficulties associated with other electrochemical methods for the determinations of reduction potentials in proteins, because equilibria are achieved rapidly and there is no interference from surface contamination of electrodes.

## 2 Materials and methods

### 2.1 Cloning of human NPAS2 and human CLOCK PAS domains

A construct of human NPAS2 (hNPAS2) corresponding to the PAS-A domain (amino acids 78–240) was prepared from a cDNA clone (Image clone # 5248433 from Source BioScience) and cloned into an *Escherichia coli* expression vector (pLEICS-07) which contains an N-terminal 6xHis-tag, an S-tag and a TEV protease cleavage site upstream of the hNPAS2 PAS-A domain. A construct of human CLOCK (hCLOCK) corresponding to the PAS-A domain (amino acids 106–265) was cloned into an *E. coli* expression vector separately (pLEICS-03), which contained an N-terminal 6xHis-tag and a TEV protease cleavage site upstream of the hCLOCK PAS-A domain, using cDNA clone (Origene). The sequencing of the hNPAS2 and hCLOCK clones was carried out by the Protein and Nucleic Acids Sequencing Laboratory (University of Leicester) and verified using an Applied Biosystems 3730 sequencer.

### 2.2 Expression and purification of hNPAS2 and hCLOCK constructs

*E. coli* Rosetta 2(DE3) cells transformed with either the hNPAS2 PAS-A/pLEICS-07 or hCLOCK PAS-A/pLEICS-03 plasmid were grown in 2× YT media containing 30 μg/mL kanamycin at 37 °C to an optical density at 600 nm of 0.6–0.8. IPTG (200 μM) was then added and the cells were incubated at 18 °C overnight. Cells were harvested by centrifugation at 5500×*g* for 20 min. Cell pellets were resuspended in lysis buffer (50 mM potassium phosphate pH 8, 300 mM potassium chloride, 10 mM imidazole) containing a protease inhibitor tablet (Roche), Lysozyme (Sigma–Aldrich) and DNAse I (Sigma–Aldrich). Magnesium sulphate was added to a final concentration of 20 mM and the cells lysed by sonication on ice at 8–10 microns for 30 s at a time, separated by 30 s intervals. The cell lysate was then centrifuged at 38 700×*g* for 50 min and the supernatant applied to a Ni-NTA column equilibrated with lysis buffer. The column was washed with 300 mL wash buffer (50 mM potassium phosphate pH 8, 300 mM potassium chloride, 20 mM imidazole) and protein eluted using 0.1 M EDTA pH 8. Concentrated protein was incubated at 4 °C overnight with TEV protease to cleave the N-terminal tags and then passed down the Ni-NTA column again to remove Tev protease and non-cleaved protein. The protein was then passed through a Ni-NTA column using wash buffer and loaded onto a Superdex75 16/60 gel filtration column equilibrated with 50 mM Tris, 50 mM potassium chloride, pH 7.5. The protein was purified in the apo form and reconstituted with heme. Peaks were observed at 413 nm and 533 nm for both hNPAS2 and CLOCK PAS-A in the UV–visible spectrum, which are similar to those seen for mouse NPAS2 PAS-A [28] and mouse CLOCK PAS-A [29]. Purity was assessed by SDS–PAGE and protein was confirmed to be the desired recombinant product by tryptic digestion followed by MALDI-ToF mass spectrometry analysis.

### 2.3 Determination of reduction potentials

Reduction potentials were determined using a buffered solution (50 mM potassium phosphate buffer, pH 7.0) containing the following constituents: xanthine as a source of electrons (30 mM stock solution, stored at −20 °C); protein (3–4 μM, to give an absorbance in a suitable range (0.1–1.0)); a suitable dye with a reduction potential close to that expected for the protein being determined (lists of suggested dyes and their reduction potentials are given in Table S1); catalase (10 mg/mL stock solution); a mixture of glucose (1 M stock solution)/glucose oxidase (175 μM stock solution), and xanthine oxidase (175 μM stock solution). In our experiments, we first add glucose (5 mM final concentration), glucose oxidase (50 μg/mL final concentration), and xanthine (300 μM final concentration) to a solution of buffer to achieve O_2_-free conditions [30]; we find that the use of glucose/glucose oxidase is effective in the removal of oxygen, in advance of adding enzyme to the solution, and is more convenient than working under nitrogen/argon atmospheres (as Massey suggested [31]) or in a glove box. Catalase (5 μg/mL final concentration) is also added to the above buffered solution to remove the hydrogen peroxide produced by the glucose oxidase. We find that freshly made solutions of catalase give more reliable results; this is especially important when measuring reduction potentials of heme enzymes with high reactivity towards peroxide, to avoid adventitious formation of ferryl heme prior to reduction of the heme to ferrous (although this problem is eliminated as soon as the solution becomes anaerobic). Protein and dye are then added to this solution, with the concentration of the dye adjusted by titration to give an absorbance which is approximately equal to that of the highest absorbance band in the protein spectra. Finally, xanthine oxidase (50 nM final concentration) was added to initiate the two-electron oxidation of xanthine to uric acid and the corresponding reduction of protein and dye. Absorbance changes corresponding to reduction of the heme were measured at a wavelength where the contribution of the dye was negligible compared to that of the protein (typically 400–420 nm, depending on the protein). Reduction of the dye was measured in the 500–700 nm region, where the largest absorbance change for the dye was observed and the change in heme absorption is negligible.

Spectra were collected (25.0 °C) every minute over a period of *ca* 40 min after the addition of xanthine oxidase (a fast scan rate is needed for optimum results and to ensure consistency of concentration determinations at different wavelengths). When spectra had remained constant for at least 10 min, sodium dithionite (an excess of *ca* 5–10 mM) was added at the end of the reaction to obtain an absorbance reading for the fully reduced protein, after which spectra were collected until the absorbance values remained constant for at least a further 5 min. Data were fitted to a Nernst plot, as derived and described in the Supplementary Material (Eq. S6). All potentials are given versus a normal hydrogen electrode (NHE).

## 3 Results

The method that we describe is a modification of that described by Massey [31]. It is conceptually and technically simplistic in the sense that spectroscopic measurements are used to obtain the concentration ratio of the oxidized and reduced partners (protein and dye), providing the equilibrium constant, which is related to potential via the Gibbs energy (using classical relationships, see Supplementary material). The method allows the determination of the reduction potential from equilibrium concentrations and without the need for direct measurement of a potential (see [32] for a recent historical perspective on early protein electrochemistry). We found that we could reliably reproduce the reduction potential of known proteins. Using toluidine blue O (*E*_m_ = 34 mV, Table S1) we determined a reduction potential for horse myoglobin (*E*_m_ = 47 mV ± 2 mV, pH 7.0, Fig. S1), which is close to the literature value at the same pH [33] (literature values for horse myoglobin vary slightly across a range of experimental conditions, reviewed in [2]). Similarly, using disodium 2,6-dichlorophenolindophenol (*E*_m_ = 217 mV, Table S1), the reduction potential of cytochrome *c* was determined as *E*_m_ = 261 mV ± 2 mV (data not shown), which compared well to literature values [34], [35], [36], [37], [38], [39]. We find that the methodology is faster than other mediated [23], [40], [41], [42] methods and more convenient than direct electrochemistry methods [16], because electrons are exchanged in bulk solution instead of via mediated electron transfer between the protein and thin-layer electrode, which means that equilibria are reached more quickly (and avoids very long equilibration times), and the process is not vulnerable to surface contamination. Since the method is based on equilibration, in principle it can be used at any pH or temperature at which the proteins involved are stable and the corresponding equilibration reactions occur at reasonable rates.^2^

**Fig. 1:**
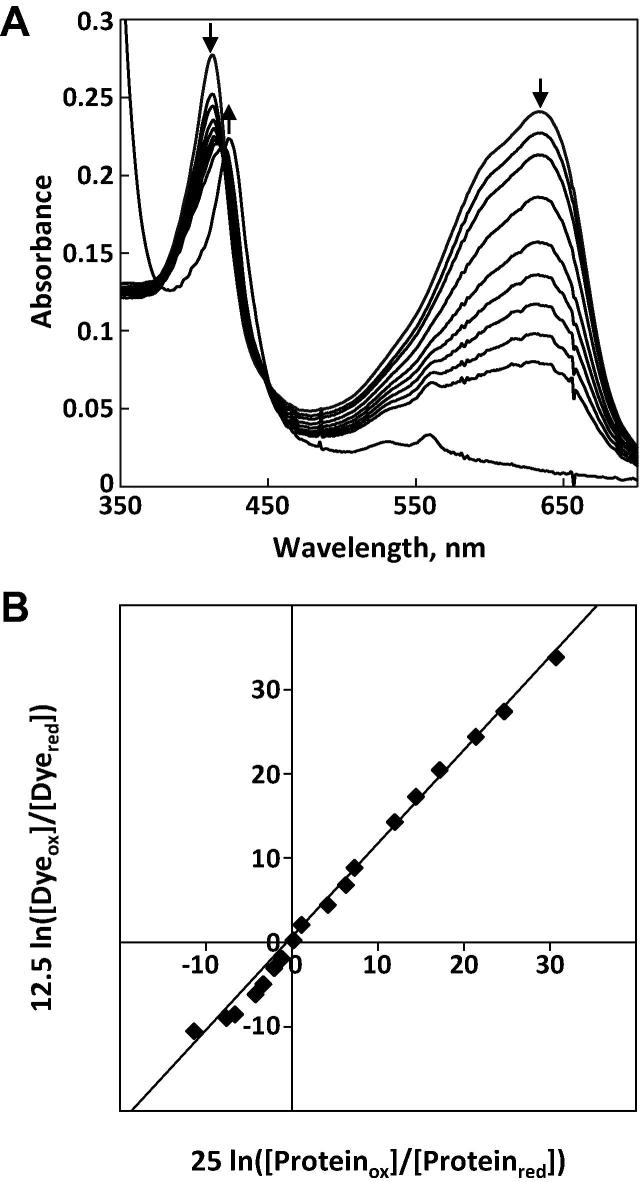
(A) Spectroscopic changes observed during the determination of the Fe^3+/2+^ reduction potential of the PAS-A domain of hNPAS2 using the dye Nile blue (25.0 °C, pH 7.0). Arrows indicate direction of absorbance changes. (B) The corresponding Nernst plot.

**Fig. 2:**
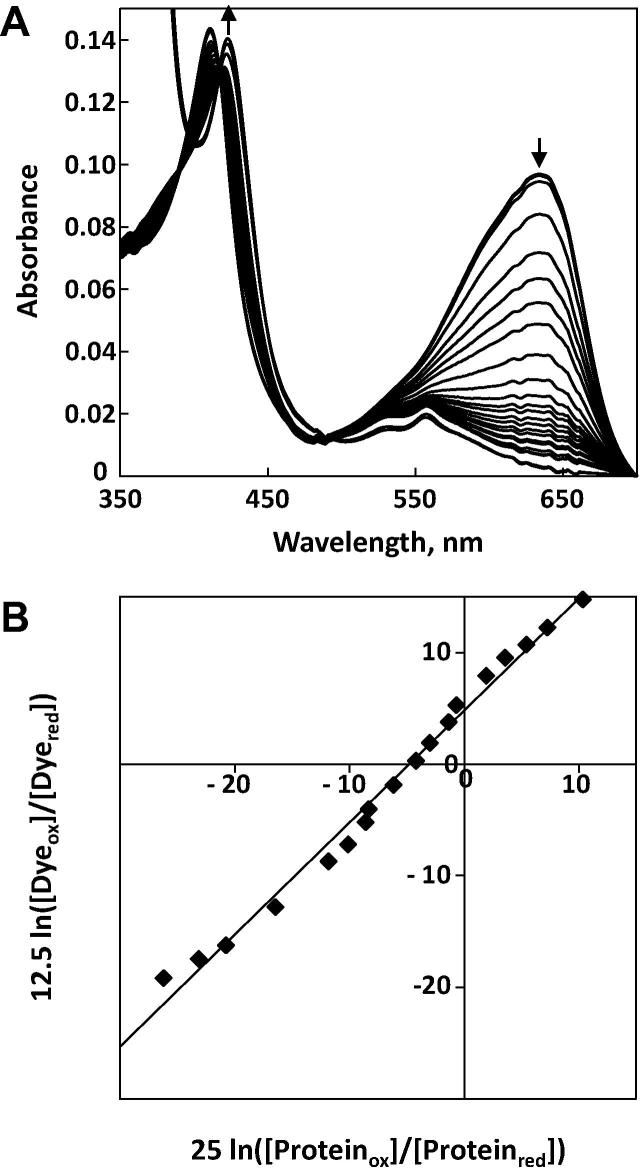
(A) Spectroscopic changes observed during the determination of the Fe^3+/2+^ reduction potential of the PAS-A domain of hCLOCK using the dye Nile blue (25.0 °C, pH 7.0). Arrows indicate direction of absorbance changes. (B) The corresponding Nernst plot.

Using the same approach, we have determined the reduction potential for two related heme-binding regulatory proteins, human NPAS2 and human CLOCK, both of which are implicated in regulation of the circadian clock [43]. NPAS2 and CLOCK form heterodimers with BMAL1 (brain and muscle Arnt-like 1) and the dimer binds to an E-box DNA sequence (CANNTG) to activate expression of the negative autoregulators, the Per and Cry genes [43]. Both NPAS2 and CLOCK contain a DNA-binding helix-loop-helix motif followed by two regulatory PAS domains (PAS-A and PAS-B), both of which are thought to bind heme [28], [44], [45]. Using Nile Blue (*E*_m_ = −116 mV, Table S1), we determined the reduction potential for the PAS-A domains of both human NPAS2 (*E*_m_ = −115 mV ± 2 mV, pH 7.0, Fig. 1) and human CLOCK (*E*_m_ = −111 mV ± 2 mV, pH 7.0, Fig. 2).

## 4 Discussion

In 1991, Massey published what was described as a simple method for the determination of redox potentials [31]. The method has since been very widely and routinely adopted in the determination of redox potentials of flavins and flavoproteins (a citation search on Web of Science finds >100 citations to the Massey paper). Although Massey himself noted the potential usefulness of this method for determinations of reduction potentials in other redox proteins, the fact that the original method focussed on one group of proteins – those containing flavin cofactors – seems to have limited its widespread adoption, because application of the method in the subsequent literature has been almost exclusively limited to flavin proteins. As we show in this paper, the method can reliably reproduce literature reduction potentials of well-known heme proteins, and we have also previously applied it to other catalytic heme enzymes [23], [46], [47]. But we also find that the method is equally well applied to other, newer heme proteins such as the regulatory heme proteins examined in this paper (which often bind heme rather more weakly). Others have recently found our approach useful [48], and we believe that the method can be widely useful for most heme proteins.

## Footnotes

2 One should, however, be aware of the drift of redox potentials of the reference dye, which depends both on temperature and on pH (see Table S1 and Ref. [1] therein).
