# Supplementary material for: A simple method for the determination of reduction potentials in heme proteins
Source: FEBS Lett. 2014 Mar 3;588(5):701–4. doi: 10.1016/j.febslet.2013.12.030 (PMC3999514; doi:10.1016/j.febslet.2013.12.030)
Supplement: Supplementary data 1 — Supplementary material. [file mmc1.docx]

**A simple method for the determination of reduction potentials in heme proteins**

Igor Efimov **^a,*^**, Gary Parkin **^a,*^**, Elizabeth S Millett **^a^**, Jennifer Glenday **^a^**, Cheuk K Chan **^a^**, Holly Weedon **^a^**, Harpreet Randhawa **^a^**, Jaswir Basran ^b^, Emma L. Raven **^a,**^**

*^a^ Department of Chemistry, Henry Wellcome Building, Uni*v*ersity of Leicester, Uni*v*ersity Road, Leicester, LE1 7RH, United Kingdom*

*^b^ Department of Biochemistry, Henry Wellcome Building, Uni*v*ersity of Leicester, Uni*v*ersity Road, Leicester, LE1 9HN, United Kingdom*

**Table S1.** Table of reduction potential for relevant dyes (pH 7.0 (1)).

| *Dye* | *E_m7_ / mV* |
| --- | --- |
| 3-bromophenol-indophenol | 248 |
| 2-chlorophenol-indophenol | 233 |
| Bindschedler’s green | 224 |
| Disodium 2,6-dichlorophenolindophenol | 217 |
| 2,6-dichlorobenzenone-indo-*o*-cresol | 181 |
| 1-Naphthol-2-sulphonate-indophenol | 123 |
| 1-Naphthol-2-sulphonate-indo-2,6-dichlorophenol | 119 |
| Toluylene blue | 115 |
| Thionine | 56 |
| Cresyl blue | 47 |
| Toluidine blue | 34 |
| Gallocyanine | 21 |
| Methylene blue | 11 |
| 5,5’,7,7’-indigotetrasulphonate | -46 |
| Methyl Capri blue | -60 |
| 5,5’,7-indigotrisulphonate | -81 |
| 5,5’-indigodisulphonate | -125 |
| Nile Blue | -116 |
| Gallophenine | -142 |
| Brilliant Alizarine blue | -173 |
| Anthraquinone-2,6-disulphonate | -184 |
| Phenosafranine | -252 |
| Tetramethylphenosafranine | -273 |
| Safranine T | -289 |
| Induline Scarlet | -299 |
| Neutral red | -325  -323 |
| Rosindone sulphonate | -385 |

**1.** All values for dyes are taken from Clark, W. M., Oxidation-Reduction Potentials of Organic Systems, Williams and Wilkin Co., Baltimore, 1960, pp. 131, 387 and 422.


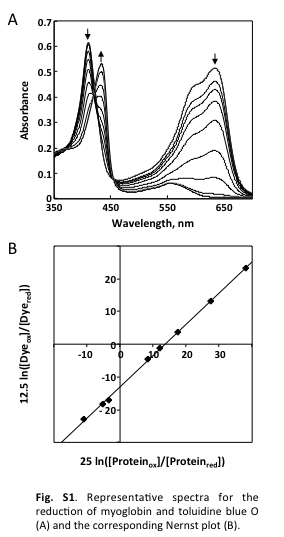


**Derivation of relevant equations**

Reduction potentials are determined using the Nernst equation (Eq. 1), where R is the gas constant, F is Faraday’s constant, n is the number of electrons transferred and T is the temperature in Kelvin. By exploiting enzymatic oxidation of xanthine it is possible to reduce both a dye (*D*), with a known mid-point potential, and the protein of interest (*P*) in solution and from this the mid-point potential of the protein can be calculated.

$$E_{P}= E_{m.P}+\frac{RT}{nF}\ln\left( \frac{\left[ P_{\mathrm{ox}} \right]}{\left[ P_{\mathrm{red}} \right]} \right) \left( \mathrm{Eq}. S1 \right)$$

Where *E*_P_ is the measured potential of the protein (*P*), and *E_m,_*_P_ is the reduction potential to be determined. It is convenient in calculations to use the product *RT*/n*F,* which is equal to 25/n mV (*i.e.* 25 mV for n = 1 (a 1-electron reduction of the protein or dye), and 12.5 mV for n = 2). Absorbance values of the protein, conveniently measured at the Soret band of the oxidised form, and the dye, measured in the visible region, over time allows determination of the ratio of concentrations of oxidised (ox) to reduced (red) form of both protein and dye at each stage of the experiment, Eq. 2^[[1]](#footnote-1)^.

$$\frac{A-A_{\min}}{A_{\max}- A}= \frac{\left[ \mathrm{oxidised} \right]}{\left[ \mathrm{reduced} \right]} \left( Eq. S2 \right)$$

Electrochemical potentials in solution are equal at equilibrium (*E*_D_ = *E*_P_). Hence, it is possible to write an expression relating absorbance changes of the dye to the reduction potential of the protein, Eq. 3 (where n = 2 for the dye and n = 1 for the protein).

$$E_{m,D}+ \frac{RT}{nF}\ln\left( \frac{\left[ D_{ox} \right]}{\left[ D_{red} \right]} \right)= E_{m,P}+ \frac{RT}{nF}\ln\left( \frac{\left[ P_{ox} \right]}{\left[ P_{red} \right]} \right) \left( Eq. S3 \right)$$

For each spectrum recorded, the Nernst concentration term for the dye (Eq. 4, where n = 2) is plotted against the Nernst concentration term for the protein (Eq. 5, where n = 1).

$$y= \frac{25}{2}\ln\left( \frac{\left[ D_{ox} \right]}{\left[ D_{red} \right]} \right) \left( Eq. S4 \right)$$

$$x = 25 ln \left( \frac{\left[ P_{ox} \right]}{\left[ P_{red} \right]} \right) \left( Eq. S5 \right)$$

If both the protein and the dye are at equilibrium between their oxidised and reduced forms when the spectrum is recorded, then Eq. 6 can be derived from Eq. 3 and the resulting plot of *y* versus *x* will be a straight line with a gradient of one and an intercept equal to *E*_m,P_ - *E*_m,D_.

$$y={(E}_{m,P}-E_{m,D})+x \left( Eq. S6 \right)$$

Knowing *E*_m,D_, the reduction potential of the protein, *E*_m,P_, can thus be deduced from the intercept which is equal to the difference between the mid-point potential of the protein and that of the dye, Eq 7.

$$\Delta E_{m}=E_{m,P}-E_{m,D} (Eq. S7)$$

Some points are routinely out of line at the beginning and end of the experiment when concentrations of oxidised:reduced are very far from 50:50 and the equilibrium is not well established. Because of this reason data points are often omitted from the start and end of the experiment until a straight line with a gradient of one is obtained.

1. Shown for the case of decreasing absorbance values. [↑](#footnote-ref-1)
